# Persistent T Cell Immunity Following Nipah Virus Infection: Evidence From Malaysian Survivors

**DOI:** 10.1093/infdis/jiag017

**Published:** 2026-01-08

**Authors:** Puteri Ainaa S Ibrahim, Hui Ming Ong, Heng Choon Cheong, Chong Tin Tan, Jie Ping Schee, Michael Selorm Avumegah, Won Fen Wong, Li-Yen Chang

**Affiliations:** Department of Medical Microbiology, Faculty of Medicine, Universiti Malaya, Kuala Lumpur, Malaysia; Department of Medical Microbiology, Faculty of Medicine, Universiti Malaya, Kuala Lumpur, Malaysia; Department of Medical Microbiology, Faculty of Medicine, Universiti Malaya, Kuala Lumpur, Malaysia; Division of Neurology, Department of Medicine, Faculty of Medicine, Universiti Malaya, Kuala Lumpur, Malaysia; Division of Neurology, Department of Medicine, Faculty of Medicine, Universiti Malaya, Kuala Lumpur, Malaysia; Laboratory Research and Innovation Department, Coalition for Epidemic Preparedness Innovation (CEPI), Oslo, Norway; Department of Medical Microbiology, Faculty of Medicine, Universiti Malaya, Kuala Lumpur, Malaysia; Department of Medical Microbiology, Faculty of Medicine, Universiti Malaya, Kuala Lumpur, Malaysia

**Keywords:** cellular immunity, immunologic memory, interferon-γ, interleukin-2, memory T cells

## Abstract

Nipah virus (NiV) induces strong humoral immunity, but the durability of NiV-specific memory T-cell responses remains unclear. We characterized cellular immunity approximately 25 years after infection in 4 survivors of the 1998 Malaysian outbreak. PBMCs were stimulated with overlapping peptide mini-pools spanning the NiV fusion (NiV-F) and attachment (NiV-G) glycoproteins, and responses were assessed using enzyme-linked immune absorbent spot and intracellular cytokine staining. Durable cytokine-producing T-cell responses were detected, with immunodominant epitopes in the NiV-G ectodomain. Survivors with neurological sequelae exhibited stronger T-cell responses and cognitive impairment, highlighting persistent cellular immunity decades after infection with implications for vaccine development.

## Teaser

Nipah virus infection elicits long-lasting T cell immunity. In 4 Malaysian survivors 25 years postinfection, durable IFN-γ and IL-2 responses to NiV-F and NiV-G peptides were detected, with immunodominant NiV-G epitopes, especially in those with neurological sequelae, informing vaccine development.

Nipah virus (NiV) is a highly pathogenic zoonotic paramyxovirus that causes severe disease in humans, with case-fatality rates exceeding 70% in some outbreaks [1, 2]. Infection triggers strong innate immune responses, including interferons and proinflammatory cytokines, which support the development of adaptive immunity [3]. However, the durability and functional quality of immunity following human NiV infection remain poorly understood. Sporadic outbreaks, high mortality, and frequent occurrence in resource-limited settings have limited opportunities for longitudinal human studies [1, 4, 5]. Although both humoral and cellular immune responses contribute to protection, correlates of long-term immunity have not been clearly defined.

Animal models demonstrate that NiV infection induces neutralizing antibodies and virus-specific T-cell responses, but translation to humans remains limited. Human studies in Malaysia and India suggest long-live antibody and T cell immunity following infection [4–6], yet the functional relevance and persistence of these responses over decades remain unclear. Given the central role of T cells in viral clearance, immune regulation, and maintenance of humoral immunity, defining long-term NiV-specific memory T-cell responses is critical for informing vaccine development. Here, we characterize the functional memory T-cell responses in 4 NiV survivors approximately 25 years postinfection (range: 24–26 years), with the aim of identifying immunodominant targets relevant for long-term protection.

## METHODS

### Study Design and Cohort

Four survivors of the 1998–1999 NiV outbreak in Kampung Sungai Nipah, Malaysia, were recruited and sampled approximately 25 years (range: 24–26 years) after infection. Prior NiV infection was confirmed by enzyme-linked immunosorbent assay and virus neutralization assays [6]. Inclusion criteria were age ≥ 18, documented NiV infection, and ability to provide informed consent. Exclusion criteria included age < 18, breastfeeding, human immunodeficiency virus/sexually transmitted infections, or refusal to consent. Two NiV-seronegative donors served as controls. Each participant underwent 6 blood collections at 2-week intervals to increase peripheral blood mononuclear cells (PBMCs) availability and assay reproducibility. Cognitive function was assessed using the Mini-Mental State Examination (MMSE) and Montreal Cognitive Assessment (MoCA) prior to sampling [7]. The study was approved by the Universiti Malaya Medical Centre Medical Research Ethics Committee (MREC ID: 202184-10454), and all participants provided written informed consent.

### PBMCs and NiV Peptides

PBMCs were isolated within 6 hours of blood collection by density-gradient centrifugation, cryopreserved in 95% fetal bovine serum and 5% dimethyl sulfoxide, and stored in liquid nitrogen vapor phase. Overlapping 15-mer peptides with 11-amino acid overlap spanning NiV fusion (NiV-F) and attachment (NiV-G) glycoproteins (Malaysia strain NV/MY/99/VRI-2794, GenBank AJ564621) were synthesized (> 85% purity). Peptides were grouped into mini-pools (F1–F9 and G1–G10), each containing 16–17 peptides, except F9 (5 peptides) and G10 (4 peptides) (Supplementary Tables 1 and 2).

### Enzyme-Linked Immune Absorbent Spot Assays

Interferon-γ (IFN-γ) and interleukin-2 (IL-2) enzyme-linked immune absorbent spot (ELISpot) assays were performed using thawed PBMCs rested overnight. Cells were stimulated with peptide mini-pools (2 µg/mL) for 48 hours. Positive controls consisted of anti-CD3-2-stimulated PBMCs; and negative controls contained unstimulated cells. Plates were scanned, and spot-forming units (S.F.U.) were quantified, background-subtracted and normalized per million cells. All assays were performed in duplicate.

### Intracellular Cytokine Staining

PBMCs were stimulated with peptide mini-pools (2 µg/mL) for 12 hours, followed by an additional 12-hour incubation in the presence of BD GolgiStop™. Phorbol 12-myristate 13-acetate and ionomycin served as positive controls. Cells were stained with viability dye, surface antibodies (CD3, CD4, CD8, CD45RA, and CCR7), fixed/permeabilized, and stained for IFN-γ and IL-2. Data were acquired on a BD FACSCanto II and analyzed using FlowJo. Responses were background-subtracted, and a ≥ 5-fold increase over solvent control was used as the threshold for positivity. Replicates were not performed because of limited cell availability.

### Sequence Conservation Analysis

Full-length NiV-G sequences from Malaysia, Bangladesh, India, and Hendra virus (HeV) sequences were retrieved from NCBI and aligned using MAFFT in Geneious Prime (v2025.1.2) (Supplementary Table 3).

### Statistical Analyses

ELISpot data were analyzed using two-way ANOVA, followed by Tukey's post hoc multiple comparison test to identify peptide mini-pools eliciting significantly stronger responses within individual participants (GraphPad Prism v10.4.2; *P* < .05).

## RESULTS

### Clinical and Cognitive Profiles

The 4 NiV survivors (S1–S4) were male, with a median age of 48.5 years (range: 37–53), at enrollment and 14–30 years old at the time of infection (Supplementary Table 4). All experienced symptomatic disease requiring hospitalization, with neurological manifestations including fever, dizziness, ataxia, and vomiting. Long-term outcomes varied. Two survivors (S2 and S3) developed persistent neurological sequelae, including ongoing seizures requiring antiepileptic therapy (S2), and long-standing visual impairment (S3). The remaining two survivors (S1 and S4) reported complete recovery.

Cognitive assessment showed normal performance in S1 and S4 (MMSE > 26; MoCA > 25). In contrast, S2 and S3 demonstrated mild to moderate cognitive impairment (MMSE 18–26; MoCA 11–25), consistent with long-term sequelae, although both remained independent in daily activities.

### NiV-Specific Memory T-Cell Responses

**Figure 1.**
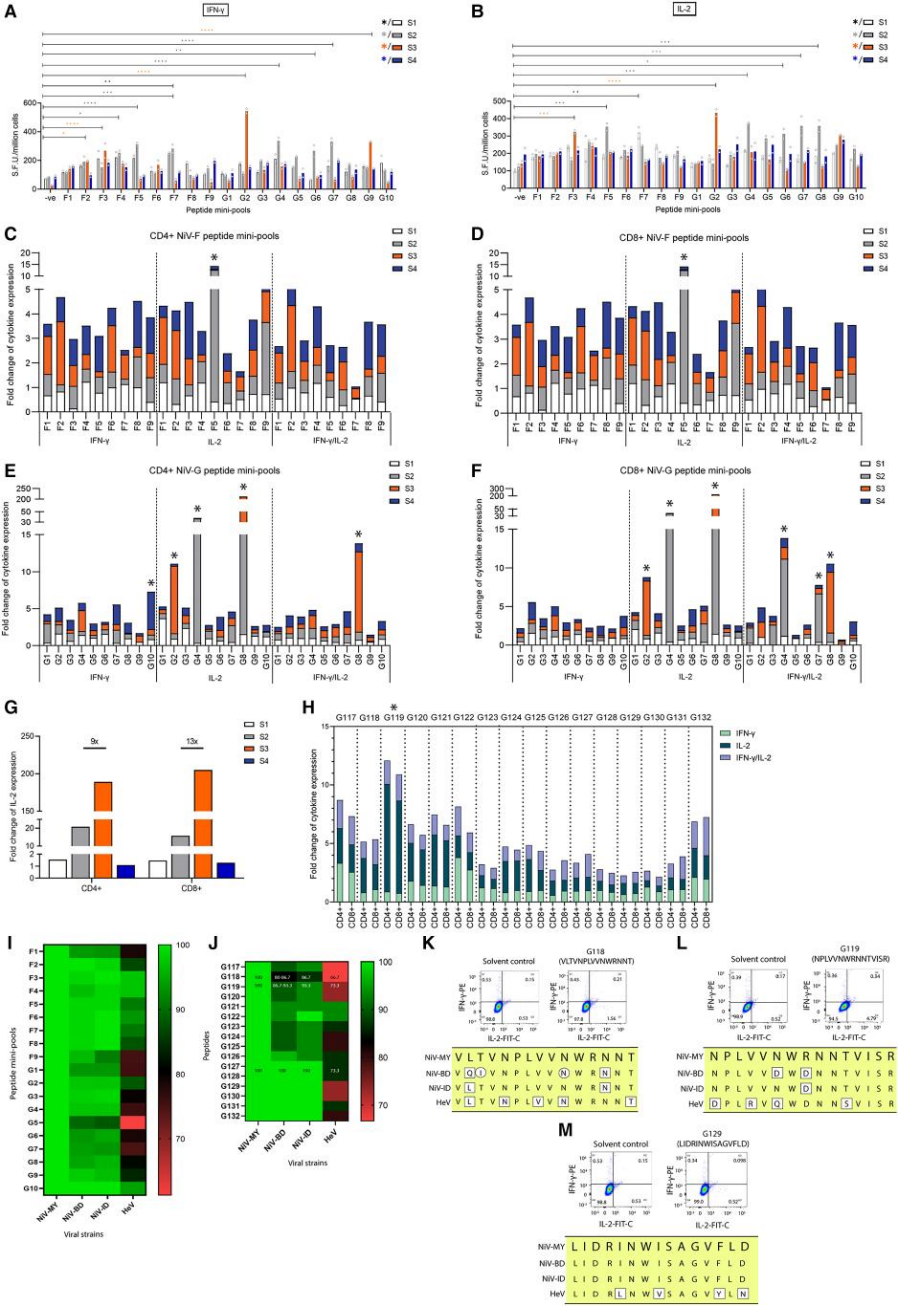
Persistent NiV-F- and NiV-G-specific memory T-cell responses and epitope conservation in long-term survivors. Memory T-cell responses to NiV-F and NiV-G peptide mini-pools measured by ELISpot. *A*, IFN-γ responses and (*B*) IL-2 responses were determined by ELISpot after stimulation of PBMCs from survivors S1, S2, S3, and S4 using NiV-F and NiV-G peptide mini-pools. Statistical significance was determined using two-way ANOVA followed by Tukey's multiple comparison post hoc test, as compared with the negative control (−ve). Asterisks denote the significance levels as *P < .05, **P < .01, ***P < .001, and ****P < .0001. Data are presented as S.F.U. per million cells. Detection of IFN-γ, IL-2, and dual cytokine (IFN-γ/IL-2) responses in CD4+ (*C*) and CD8+ (*D*) T cells from NiV survivors S1, S2, S3, and S4 stimulated with NiV-F peptide mini-pools, and comparison of cytokine production in CD4+ (*E*) and CD8+ (*F*) T cells stimulated with NiV-G peptide mini-pools. The peptide mini-pools that induced cytokine production by ≥ 5-fold compared with solvent controls in both CD4+ and CD8+ T cell subsets are marked with an asterisk. *G*, Comparison of IL-2 expression levels in CD4+ and CD8+ T cells from NiV survivors S1, S2, S3, and S4 after stimulation with the G8 peptide mini-pool. The horizontal line between S2 and S3 indicates the fold change difference between the samples compared with solvent control. *H*, Analysis of IFN-γ, IL-2, and dual cytokine (IFN-γ/IL-2) responses in CD4+ and CD8+ T cells by ICS for NiV survivor S3, stimulated with single peptides from the G8 peptide mini-pool. The peptide that induced cytokine production by ≥ 5-fold compared with solvent controls in both CD4+ and CD8+ T cells is marked with an asterisk. *I*, Heat map depicting the percentage sequence identity of all NiV peptide mini-pools compared with NiV-M, NiV-BD, NiV-ID, and HeV. *J*, Heat map showing sequence conservation of individual peptides within the G8 peptide mini-pool across the same viral strains. Detailed percentage identity is provided for three peptides, G118, G119, and G129. Representative ICS dot plots of CD4+ T-cell responses for survivor S3 following stimulation with three peptides from the G8 peptide mini-pool: (*K*) G118 (moderately immunogenic, lowest conservation), (*L*) G119 (highly immunogenic, moderate-to-high conservation), and (*M*) G129 (low immunogenicity, high conservation). Solvent controls are shown for comparison. Sequence variations are annotated as follows: squares (□) indicate positions with a consistent amino acid substitution across all sequences of a given strain; circles (○) indicate variable substitutions observed in only some sequences of that strain.

ELISpot assays detected IFN-γ and/or IL-2 responses in 3 of 4 survivors (S1–S3), with the strongest responses observed in S2 and S3 (Figure 1*A* and 1*B*). Significant IFN-γ responses to NiV-F peptide mini-pools were detected in S1 (F7), S2 (F4, F5, F7), and S3 (F2, F3). NiV-G mini-pools induced significant IFN-γ responses in S2 (G4, G6, G7) and S3 (G2, G9). IL-2 responses were more restricted but significant in S1 which responded to F7, S2 to F5, S3 to F3. Within NiV-G mini-pools, significant respond was prominent in S2 and S3, particularly to G4, G6, G7, and G8 in S2, and to G2 in S3. No significant ELISpot responses were observed in S4.

Intracellular cytokine staining (ICS) confirmed these findings and identified responding T cell subsets (Figure 1*C*–*F*). Survivors S2 and S3 exhibited robust IL-2 production in both CD4+ and CD8+ T cells, with detectable dual IFN-γ/IL-2-producing populations. S4, despite lacking detectable ELISpot responses, showed a CD4+ IFN-γ responses to G10 mini-pool.

### Identification of Immunogenic Peptide Mini-Pools and Epitopes

Across assays, peptide mini-pools F5, G2, G4, and G8 elicited reproducible cytokine responses in at least 1 survivor (Table 1). Among these, G8 was the most prominent, inducing strong polyfunctional responses, particularly in survivor S3. In S2, G8 induced a > 10-fold increase in IL-2 in both subsets, while S3 showed a 9-fold increase in CD4+ and a 13-fold increase in CD8+ T cells compared with S2 (Figure 1*G*). Subsequent ICS analysis of individual peptides within the G8 mini-pool in survivor S3 identified peptide G119 as the immunodominant epitope, inducing > 5-fold IL-2 responses in both CD4+ and CD8+ T cells (Figure 1*H*). Several additional peptides within G8 elicited intermediate responses, whereas others were minimally immunogenic.

**Table 1.** IFN-γ and IL-2 Responses to NiV-F and NiV-G Peptide Mini-pools in NiV Survivors

Assay	NiV Survivor	Cytokine Expression		Peptide Mini-Pool																		
				F1	F2	F3	F4	F5	F6	F7	F8	F9	G1	G2	G3	G4	G5	G6	G7	G8	G9	G10
ELISpot^a^	S1	IFN-γ		…	…	…	…	…	…	+	…	…	…	…	…	…	…	…	…	…	…	…
		IL-2		…	…	…	…	…	…	+	…	…	…	…	…	…	…	…	…	…	…	…
	S2	IFN-γ		…	…	…	+	+	…	+	…	…	…	…	…	+	…	+	+	…	…	…
		IL-2		…	…	…	…	+	…	…	…	…	…	…	…	+	…	+	+	+	…	…
	S3	IFN-γ		…	+	+	…	…	…	…	…	…	…	+	…	…	…	…	…	…	+	…
		IL-2		…	…	+	…	…	…	…	…	…	…	+	…	…	…	…	…	…	…	…
	S4	IFN-γ		…	…	…	…	…	…	…	…	…	…	…	…	…	…	…	…	…	…	…
		IL-2		…	…	…	…	…	…	…	…	…	…	…	…	…	…	…	…	…	…	…
ICS^b^	S1	IFN-γ	CD4+	…	…	…	…	…	…	…	…	…	…	…	…	…	…	…	…	…	…	…
			CD8+	…	…	…	…	…	…	…	…	…	…	…	…	…	…	…	…	…	…	…
		IL-2	CD4+	…	…	…	…	…	…	…	…	…	…	…	…	…	…	…	…	…	…	…
			CD8+	…	…	…	…	…	…	…	…	…	…	…	…	…	…	…	…	…	…	…
		IFN-γ/IL-2	CD4+	…	…	…	…	…	…	…	…	…	…	…	…	…	…	…	…	…	…	…
			CD8+	…	…	…	…	…	…	…	…	…	…	…	…	…	…	…	…	…	…	…
	S2	IFN-γ	CD4+	…	…	…	…	…	…	…	…	…	…	…	…	…	…	…	…	…	…	…
			CD8+	…	…	…	…	…	…	…	…	…	…	…	…	…	…	…	…	…	…	…
		IL-2	CD4+	…	…	…	…	+	…	…	…	…	…	…	…	+	…	…	…	+	…	…
			CD8+	…	…	…	…	+	…	…	…	…	…	…	…	+	…	…	…	+	…	…
		IFN-γ/IL-2	CD4+	…	…	…	…	…	…	…	…	…	…	…	…	…	…	…	…	…	…	…
			CD8+	…	…	…	…	…	…	…	…	…	…	…	…	+	…	…	+	…	…	…
	S3	IFN-γ	CD4+	…	…	…	…	…	…	…	…	…	…	…	…	…	…	…	…	…	…	…
			CD8+	…	…	…	…	…	…	…	…	…	…	…	…	…	…	…	…	…	…	…
		IL-2	CD4+	…	…	…	…	…	…	…	…	…	…	+	…	…	…	…	…	+	…	…
			CD8+	…	…	…	…	…	…	…	…	…	…	+	…	…	…	…	…	+	…	…
		IFN-γ/IL-2	CD4+	…	…	…	…	…	…	…	…	…	…	…	…	…	…	…	…	+	…	…
			CD8+	…	…	…	…	…	…	…	…	…	…	…	…	…	…	…	…	+	…	…
	S4	IFN-γ	CD4+	…	…	…	…	…	…	…	…	…	…	…	…	…	…	…	…	…	…	+
			CD8+	…	…	…	…	…	…	…	…	…	…	…	…	…	…	…	…	…	…	…
		IL-2	CD4+	…	…	…	…	…	…	…	…	…	…	…	…	…	…	…	…	…	…	…
			CD8+	…	…	…	…	…	…	…	…	…	…	…	…	…	…	…	…	…	…	…
		IFN-γ/IL-2	CD4+	…	…	…	…	…	…	…	…	…	…	…	…	…	…	…	…	…	…	…
			CD8+	…	…	…	…	…	…	…	…	…	…	…	…	…	…	…	…	…	…	…

^a^In the ELISpot assay, a (+) symbol indicates a statistically significant difference from the negative control, with *P* values ranging from .05 to .0001.

^b^In the ICS assay, a (+) symbol indicates a positive response to peptide mini-pools, defined as a stimulation of ≥ 5-fold increase compared with the solvent control.

### Sequence Conservation

Sequence alignment revealed high conservation of immunogenic peptide regions across NiV strains from Malaysia, Bangladesh, and India, with moderate conservation relative to HeV (Figure 1*I*). The G8 mini-pool, including the immunodominant peptide G119, retained substantial sequence identity across NiV lineages and partial conservation with HeV (Figure 1*J*) and induced robust IL-2 production (Figure 1*L*). However, sequence conservation alone did not uniformly predict immunogenicity (Figure 1*K* and 1*M*).

## DISCUSSION

This study demonstrates that NiV-specific memory T-cell responses can persist for at least 25 years after natural infection. This represents the longest documented persistence of functional cellular immunity following NiV infection in humans. Despite the prolonged interval since exposure, 3 or 4 survivors exhibited measurable, antigen-specific T-cell responses, underscoring the remarkable durability of NiV-induced cellular immunity.

Interindividual heterogeneity was a prominent feature of the immune responses observed. Survivors with persistent neurological sequelae (S2 and S3) mounted the strongest and most polyfunctional T-cell responses, whereas those with complete recovery showed weaker or undetectable responses by ELISpot. Although the small cohort precludes statistical inference, this pattern parallels observations from other viral infections, including severe acute respiratory syndrome coronavirus 1 (SARS-CoV-1), SARS-CoV-2, and Ebola virus, in which greater disease severity has been associated with durable immune memory [8–10]. One plausible explanation is that higher viral burden or prolonged antigen exposure during acute neuroinvasive disease drives more robust long-term T cell persistence. Alternatively, sustained immune activation may contribute to chronic neurological sequelae through immune-mediated mechanisms. Distinguishing protective from potentially pathogenic memory responses will require larger, longitudinal studies integrating immunological and neurological outcomes.

The predominance of IL-2 producing memory T cells decades after infection is noteworthy. IL-2 production is characteristic of central memory T cells with proliferative capacity and long-term survival potential. The persistence of IL-2-dominant responses suggests that NiV-specific memory T cells remain functionally competent rather than terminally differentiated or exhausted. This phenotype is desirable for vaccine-induced immunity, as IL-2 supports recall expansion and coordination of humoral immune responses [11].

NiV-G emerged as a major target of long-term T cell immunity. The identification of conserved, immunodominant epitopes within the NiV-G ectodomain, particularly peptide G119, has important implications for vaccine development. Current vaccine candidates, including subunit and mRNA-based platforms, focus on NiV-G because of its role in receptor binding and induction of neutralizing antibodies [12, 13]. Our findings indicate that NiV-G also elicits durable CD4+ and CD8+ T-cell responses, supporting its inclusion as a dual-function antigen capable of engaging both humoral and cellular immunity.

Sequence conservation analysis further supports the translational relevance of these findings. Several immunogenic regions were highly conserved across NiV strains from Malaysia, Bangladesh, and India, suggesting potential for cross-lineage protection. However, not all conserved peptides were immunogenic, indicating that antigen processing, human leukocyte activation (HLA) restriction, and epitope accessibility play critical roles in shaping T cell recognition [14]. These observations highlight the importance of empirical epitope mapping in humans, particularly for pathogens with pandemic potential.

This study has limitations, including the small cohort size of 4 survivors, restricted HLA diversity, cross-sectional design, and focus on a limited set of cytokines, which necessitate caution in generalizing the findings. Nonetheless, it provides rare insight into the exceptional longevity of cellular immunity following NiV infection. The heterogeneity of responses observed underscores the need for larger studies across diverse geographic settings and viral lineages, including Bangladesh and India. These findings highlight the importance of long-term immunological follow-up of NiV case-patients from acute infection through long-term recovery.

In conclusion, NiV-specific memory T-cell responses persist for decades after infection and preferentially target conserved regions of the viral attachment glycoprotein. These findings advance our understanding of long-term immunity to NiV and provide a rationale for vaccine strategies aimed at inducing durable, polyfunctional T-cell responses against conserved henipavirus antigens.

## Supplementary Material

jiag017_Supplementary_Data

## Supplementary Data

Supplementary materials are available at *The Journal of Infectious Diseases* online (http://jid.oxfordjournals.org/). Supplementary materials consist of data provided by the author that are published to benefit the reader. The posted materials are not copyedited. The contents of all supplementary data are the sole responsibility of the authors. Questions or messages regarding errors should be addressed to the author.
