# Supplementary material for: Persistent T Cell Immunity Following Nipah Virus Infection: Evidence From Malaysian Survivors
Source: J Infect Dis. 2026 Jan 8;233(5):e1261–6. doi: 10.1093/infdis/jiag017 (PMC13175623; doi:10.1093/infdis/jiag017)
Supplement: jiag017_Supplementary_Data [file jiag017_supplementary_data.docx]

**Supplementary Table 1. NiV-F Peptide Libraries**

| **NiV-F** | | | | | | | | | | | | |
| --- | --- | --- | --- | --- | --- | --- | --- | --- | --- | --- | --- | --- |
| **F1** | | | **F2** | | | **F3** | | | | **F4** | | |
| **Peptide number** | **Peptide sequence** | **aa^a^** | **Peptide number** | **Peptide sequence** | **aa^a^** | **Peptide number** | **Peptide sequence** | **aa^a^** | **Peptide number** | | **Peptide sequence** | **aa^a^** |
| 1 | MVVILDKRCYCNLLI | 1–15 | 18 | SQCTGSVMENYKTRL | 69–83 | 36 | ADNINKLKSSIESTN | 141–155 | 52 | | KYLSDLLFVFGPNLQ | 205–219 |
| 2 | LDKRCYCNLLILILM | 5–19 | 19 | GSVMENYKTRLNGIL | 73–87 | 37 | NKLKSSIESTNEAVV | 145–159 | 53 | | DLLFVFGPNLQDPVS | 209–223 |
| 4 | LLILILMISECSVGI | 13–27 | 20 | ENYKTRLNGILTPIK | 77–91 | 38 | SSIESTNEAVVKLQE | 149–163 | 54 | | VFGPNLQDPVSNSMT | 213–227 |
| 5 | ILMISECSVGILHYE | 17–31 | 21 | TRLNGILTPIKGALE | 81–95 | 39 | STNEAVVKLQETAEK | 153–167 | 55 | | NLQDPVSNSMTIQAI | 217–231 |
| 6 | SECSVGILHYEKLSK | 21–35 | 22 | GILTPIKGALEIYKN | 85–99 | 40 | AVVKLQETAEKTVYV | 157–171 | 56 | | PVSNSMTIQAISQAF | 221–235 |
| 7 | VGILHYEKLSKIGLV | 25–39 | 23 | PIKGALEIYKNNTHD | 89–103 | 41 | LQETAEKTVYVLTAL | 161–175 | 57 | | SMTIQAISQAFGGNY | 225–239 |
| 8 | HYEKLSKIGLVKGVT | 29–43 | 24 | ALEIYKNNTHDLVGD | 93–107 | 42 | AEKTVYVLTALQDYI | 165–179 | 58 | | QAISQAFGGNYETLL | 229–243 |
| 9 | LSKIGLVKGVTRKYK | 33–47 | 25 | YKNNTHDLVGDVRLA | 97–111 | 43 | VYVLTALQDYINTNL | 169–183 | 59 | | QAFGGNYETLLRTLG | 233–247 |
| 10 | GLVKGVTRKYKIKSN | 37–51 | 26 | THDLVGDVRLAGVIM | 101–115 | 44 | TALQDYINTNLVPTI | 173–187 | 60 | | GNYETLLRTLGYATE | 237–251 |
| 11 | GVTRKYKIKSNPLTK | 41–55 | 27 | VGDVRLAGVIMAGVA | 105–119 | 45 | DYINTNLVPTIDKIS | 177–191 | 61 | | TLLRTLGYATEDFDD | 241–255 |
| 12 | KYKIKSNPLTKDIVI | 45–59 | 29 | VIMAGVAIGIATAAQ | 113–127 | 46 | TNLVPTIDKISCKQT | 181–195 | 62 | | TLGYATEDFDDLLES | 245–259 |
| 13 | KSNPLTKDIVIKMIP | 49–63 | 31 | GIATAAQITAGVALY | 121–135 | 47 | PTIDKISCKQTELSL | 185–199 | 63 | | ATEDFDDLLESDSIT | 249–263 |
| 14 | LTKDIVIKMIPNVSN | 53–67 | 32 | AAQITAGVALYEAMK | 125–139 | 48 | KISCKQTELSLDLAL | 189–203 | 64 | | FDDLLESDSITGQII | 253–267 |
| 15 | IVIKMIPNVSNMSQC | 57–71 | 33 | TAGVALYEAMKNADN | 129–143 | 49 | KQTELSLDLALSKYL | 193–207 | 65 | | LESDSITGQIIYVDL | 257–271 |
| 16 | MIPNVSNMSQCTGSV | 61–75 | 34 | ALYEAMKNADNINKL | 133–147 | 50 | LSLDLALSKYLSDLL | 197–211 | 66 | | SITGQIIYVDLSSYY | 261–275 |
| 17 | VSNMSQCTGSVMENY | 65–79 | 35 | AMKNADNINKLKSSI | 137–151 | 51 | LALSKYLSDLLFVFG | 201–215 | 67 | | QIIYVDLSSYYIIVR | 265–279 |

^a^‘aa’ refers to amino acid.

| **NiV-F** | | | | | | | | | | | |
| --- | --- | --- | --- | --- | --- | --- | --- | --- | --- | --- | --- |
| **F5** | | | **F6** | | | **F7** | | | **F8** | | |
| **Peptide number** | **Peptide sequence** | **aa^a^** | **Peptide number** | **Peptide sequence** | **aa^a^** | **Peptide number** | **Peptide sequence** | **aa^a^** | **Peptide number** | **Peptide sequence** | **aa^a^** |
| 68 | VDLSSYYIIVRVYFP | 269–283 | 85 | SVICNQDYATPMTNN | 337–351 | 101 | ISQSGEQTLLMIDNT | 401–415 | 117 | QSLQQSKDYIKEAQR | 465–479 |
| 69 | SYYIIVRVYFPILTE | 273–287 | 86 | NQDYATPMTNNMREC | 341–355 | 102 | GEQTLLMIDNTTCPT | 405–419 | 118 | QSKDYIKEAQRLLDT | 469–483 |
| 70 | IVRVYFPILTEIQQA | 277–291 | 87 | ATPMTNNMRECLTGS | 345–359 | 103 | LLMIDNTTCPTAVLG | 409–423 | 119 | YIKEAQRLLDTVNPS | 473–487 |
| 71 | YFPILTEIQQAYIQE | 281–295 | 88 | TNNMRECLTGSTEKC | 349–363 | 104 | DNTTCPTAVLGNVII | 413–427 | 120 | AQRLLDTVNPSLISM | 477–491 |
| 72 | LTEIQQAYIQELLPV | 285–299 | 89 | RECLTGSTEKCPREL | 353–367 | 105 | CPTAVLGNVIISLGK | 417–431 | 121 | LDTVNPSLISMLSMI | 481–495 |
| 73 | QQAYIQELLPVSFNN | 289–303 | 90 | TGSTEKCPRELVVSS | 357–371 | 106 | VLGNVIISLGKYLGS | 421–435 | 122 | NPSLISMLSMIILYV | 485–499 |
| 74 | IQELLPVSFNNDNSE | 293–307 | 91 | EKCPRELVVSSHVPR | 361–375 | 107 | VIISLGKYLGSVNYN | 425–439 | 124 | SMIILYVLSIASLCI | 493–507 |
| 75 | LPVSFNNDNSEWISI | 297–311 | 92 | RELVVSSHVPRFALS | 365–379 | 108 | LGKYLGSVNYNSEGI | 429–443 | 125 | LYVLSIASLCIGLIT | 497–511 |
| 76 | FNNDNSEWISIVPNF | 301–315 | 93 | VSSHVPRFALSNGVL | 369–383 | 109 | LGSVNYNSEGIAIGP | 433–447 | 126 | SIASLCIGLITFISF | 501–515 |
| 77 | NSEWISIVPNFILVR | 305–319 | 94 | VPRFALSNGVLFANC | 373–387 | 110 | NYNSEGIAIGPPVFT | 437–451 | 127 | LCIGLITFISFIIVE | 505–519 |
| 78 | ISIVPNFILVRNTLI | 309–323 | 95 | ALSNGVLFANCISVT | 377–391 | 111 | EGIAIGPPVFTDKVD | 441–455 | 128 | LITFISFIIVEKKRN | 509–523 |
| 79 | PNFILVRNTLISNIE | 313–327 | 96 | GVLFANCISVTCQCQ | 381–395 | 112 | IGPPVFTDKVDISSQ | 445–459 | 129 | ISFIIVEKKRNTYSR | 513–527 |
| 80 | LVRNTLISNIEIGFC | 317–331 | 97 | ANCISVTCQCQTTGR | 385–399 | 113 | VFTDKVDISSQISSM | 449–463 | 130 | IVEKKRNTYSRLEDR | 517–531 |
| 82 | NIEIGFCLITKRSVI | 325–339 | 98 | SVTCQCQTTGRAISQ | 389–403 | 114 | KVDISSQISSMNQSL | 453–467 | 131 | KRNTYSRLEDRRVRP | 521–535 |
| 83 | GFCLITKRSVICNQD | 329–343 | 99 | QCQTTGRAISQSGEQ | 393–407 | 115 | SSQISSMNQSLQQSK | 457–471 | 132 | YSRLEDRRVRPTSSG | 525–539 |
| 84 | ITKRSVICNQDYATP | 333–347 | 100 | TGRAISQSGEQTLLM | 397–411 | 116 | SSMNQSLQQSKDYIK | 461–475 | 133 | EDRRVRPTSSGDLYY | 529–543 |
|  |  |  |  |  |  |  |  |  | 134 | VRPTSSGDLYYIGT | 533–546 |

^a^‘aa’ refers to amino acid.

| **NiV-F** | | |
| --- | --- | --- |
| **F9** | | |
| **Peptide number** | **Peptide sequence** | **aa^a^** |
| 3 | CYCNLLILILMISEC | 9–23 |
| 28 | RLAGVIMAGVAIGIA | 109–123 |
| 30 | GVAIGIATAAQITAG | 117–131 |
| 81 | TLISNIEIGFCLITK | 321–335 |
| 123 | ISMLSMIILYVLSIA | 489–503 |

^a^‘aa’ refers to amino acid.**Supplementary Table 2. NiV-G Peptide Libraries**

| **NiV-G** | | | | | | | | | | | | |
| --- | --- | --- | --- | --- | --- | --- | --- | --- | --- | --- | --- | --- |
| **G1** | | | **G2** | | | **G3** | | | | **G4** | | |
| **Peptide number** | **Peptide sequence** | **aa^a^** | **Peptide number** | **Peptide sequence** | **aa^a^** | | **Peptide number** | **Peptide sequence** | **aa^a^** | **Peptide number** | **Peptide sequence** | **aa^a^** |
| 1 | MPAENKKVRFENTTS | 1–15 | 21 | AVIKDALQGIQQQIK | 81–95 | | 37 | KCKFTLPPLKIHECN | 145–159 | 53 | VVGQSGTCITDPLLA | 209–223 |
| 2 | NKKVRFENTTSDKGK | 5–19 | 22 | DALQGIQQQIKGLAD | 85–99 | | 38 | TLPPLKIHECNISCP | 149–163 | 54 | SGTCITDPLLAMDEG | 213–227 |
| 3 | RFENTTSDKGKIPSK | 13–27 | 23 | GIQQQIKGLADKIGT | 89–103 | | 39 | LKIHECNISCPNPLP | 153–167 | 55 | ITDPLLAMDEGYFAY | 217–231 |
| 4 | TTSDKGKIPSKVIKS | 17–31 | 24 | QIKGLADKIGTEIGP | 93–107 | | 40 | ECNISCPNPLPFREY | 157–171 | 56 | LLAMDEGYFAYSHLE | 221–235 |
| 5 | KGKIPSKVIKSYYGT | 21–35 | 25 | LADKIGTEIGPKVSL | 97–111 | | 41 | SCPNPLPFREYRPQT | 161–175 | 57 | DEGYFAYSHLERIGS | 225–239 |
| 6 | PSKVIKSYYGTMDIK | 25–39 | 26 | IGTEIGPKVSLIDTS | 101–115 | | 42 | PLPFREYRPQTEGVS | 165–179 | 58 | FAYSHLERIGSCSRG | 229–243 |
| 7 | IKSYYGTMDIKKINE | 29–43 | 27 | IGPKVSLIDTSSTIT | 105–119 | | 43 | REYRPQTEGVSNLVG | 169–183 | 59 | HLERIGSCSRGVSKQ | 233–247 |
| 8 | YGTMDIKKINEGLLD | 33–47 | 28 | VSLIDTSSTITIPAN | 109–123 | | 44 | PQTEGVSNLVGLPNN | 173–187 | 60 | IGSCSRGVSKQRIIG | 237–251 |
| 9 | DIKKINEGLLDSKIL | 37–51 | 29 | DTSSTITIPANIGLL | 113–127 | | 45 | GVSNLVGLPNNICLQ | 177–191 | 61 | SRGVSKQRIIGVGEV | 241–255 |
| 10 | INEGLLDSKILSAFN | 1–15 | 30 | TITIPANIGLLGSKI | 121–135 | | 46 | LVGLPNNICLQKTSN | 181–195 | 62 | SKQRIIGVGEVLDRG | 245–259 |
| 12 | KILSAFNTVIALLGS | 45–59 | 31 | PANIGLLGSKISQST | 125–139 | | 47 | PNNICLQKTSNQILK | 185–199 | 63 | IIGVGEVLDRGDEVP | 249–263 |
| 13 | AFNTVIALLGSIVII | 49–63 | 32 | GLLGSKISQSTASIN | 129–143 | | 48 | CLQKTSNQILKPKLI | 189–203 | 64 | GEVLDRGDEVPSLFM | 253–267 |
| 17 | MNIMIIQNYTRSTDN | 65–79 | 33 | SKISQSTASINENVN | 133–147 | | 49 | TSNQILKPKLISYTL | 193–207 | 65 | DRGDEVPSLFMTNVW | 257–271 |
| 18 | IIQNYTRSTDNQAVI | 69–83 | 34 | QSTASINENVNEKCK | 137–151 | | 50 | ILKPKLISYTLPVVG | 197–211 | 66 | EVPSLFMTNVWTPPN | 261–275 |
| 19 | YTRSTDNQAVIKDAL | 73–87 | 35 | SINENVNEKCKFTLP | 121–135 | | 51 | KLISYTLPVVGQSGT | 201–215 | 67 | LFMTNVWTPPNPNTV | 265–279 |
| 20 | TDNQAVIKDALQGIQ | 77–91 | 36 | NVNEKCKFTLPPLKI | 141–155 | | 52 | YTLPVVGQSGTCITD | 205–219 | 68 | NVWTPPNPNTVYHCS | 269–283 |

^a^‘aa’ refers to amino acid.

| **NiV-G** | | | | | | | | | | | |
| --- | --- | --- | --- | --- | --- | --- | --- | --- | --- | --- | --- |
| **G5** | | | **G6** | | | **G7** | | | **G8** | | |
| **Peptide number** | **Peptide sequence** | **aa^a^** | **Peptide number** | **Peptide sequence** | **aa^a^** | **Peptide number** | **Peptide sequence** | **aa^a^** | **Peptide number** | **Peptide sequence** | **aa^a^** |
| 69 | PPNPNTVYHCSAVYN | 273–287 | 85 | LRSIEKGRYDKVMPY | 337–351 | 101 | IRPNSHYILRSGLLK | 401–415 | 117 | KFGDVLTVNPLVVNW | 465–479 |
| 70 | NTVYHCSAVYNNEFY | 277–291 | 86 | EKGRYDKVMPYGPSG | 341–355 | 102 | SHYILRSGLLKYNLS | 405–419 | 118 | VLTVNPLVVNWRNNT | 469–483 |
| 71 | HCSAVYNNEFYYVLC | 281–295 | 87 | YDKVMPYGPSGIKQG | 345–359 | 103 | LRSGLLKYNLSDGEN | 409–423 | 119 | NPLVVNWRNNTVISR | 473–487 |
| 72 | VYNNEFYYVLCAVST | 285–299 | 88 | MPYGPSGIKQGDTLY | 349–363 | 104 | LLKYNLSDGENPKVV | 413–427 | 120 | VNWRNNTVISRPGQS | 477–491 |
| 73 | EFYYVLCAVSTVGDP | 289–303 | 89 | PSGIKQGDTLYFPAV | 353–367 | 105 | NLSDGENPKVVFIEI | 417–431 | 121 | NNTVISRPGQSQCPR | 481–495 |
| 74 | VLCAVSTVGDPILNS | 293–307 | 90 | KQGDTLYFPAVGFLV | 357–371 | 106 | GENPKVVFIEISDQR | 421–435 | 122 | ISRPGQSQCPRFNTC | 485–499 |
| 75 | VSTVGDPILNSTYWS | 297–311 | 91 | TLYFPAVGFLVRTEF | 361–375 | 107 | KVVFIEISDQRLSIG | 425–439 | 123 | GQSQCPRFNTCPEIC | 489–503 |
| 76 | GDPILNSTYWSGSLM | 301–315 | 92 | PAVGFLVRTEFKYND | 365–379 | 108 | IEISDQRLSIGSPSK | 429–443 | 124 | CPRFNTCPEICWEGV | 493–507 |
| 77 | LNSTYWSGSLMMTRL | 305–319 | 93 | FLVRTEFKYNDSNCP | 369–383 | 109 | DQRLSIGSPSKIYDS | 433–447 | 125 | NTCPEICWEGVYNDA | 497–511 |
| 78 | YWSGSLMMTRLAVKP | 309–323 | 94 | TEFKYNDSNCPITKC | 373–387 | 110 | SIGSPSKIYDSLGQP | 437–451 | 126 | EICWEGVYNDAFLID | 501–515 |
| 79 | SLMMTRLAVKPKSNG | 313–327 | 95 | YNDSNCPITKCQYSK | 377–391 | 111 | PSKIYDSLGQPVFYQ | 441–455 | 127 | EGVYNDAFLIDRINW | 505–519 |
| 80 | TRLAVKPKSNGGGYN | 317–331 | 96 | NCPITKCQYSKPENC | 381–395 | 112 | YDSLGQPVFYQASFS | 445–459 | 128 | NDAFLIDRINWISAG | 509–523 |
| 81 | VKPKSNGGGYNQHQL | 321–335 | 97 | TKCQYSKPENCRLSM | 385–399 | 113 | GQPVFYQASFSWDTM | 449–463 | 129 | LIDRINWISAGVFLD | 513–527 |
| 82 | SNGGGYNQHQLALRS | 325–339 | 98 | YSKPENCRLSMGIRP | 389–403 | 114 | FYQASFSWDTMIKFG | 453–467 | 130 | INWISAGVFLDSNQT | 517–531 |
| 83 | GYNQHQLALRSIEKG | 329–343 | 99 | ENCRLSMGIRPNSHY | 393–407 | 115 | SFSWDTMIKFGDVLT | 457–471 | 131 | SAGVFLDSNQTAENP | 521–535 |
| 84 | HQLALRSIEKGRYDK | 333–347 | 100 | LSMGIRPNSHYILRS | 397–411 | 116 | DTMIKFGDVLTVNPL | 461–475 | 132 | FLDSNQTAENPVFTV | 525–539 |

^a^‘aa’ refers to amino acid.

| **NiV-G** | | | | | |
| --- | --- | --- | --- | --- | --- |
| **G9** | | | **G10** | | |
| **Peptide number** | **Peptide sequence** | **aa^a^** | **Peptide number** | **Peptide sequence** | **aa^a^** |
| 133 | NQTAENPVFTVFKDN | 529–543 | 11 | LLDSKILSAFNTVIA | 41–55 |
| 134 | ENPVFTVFKDNEILY | 533–546 | 14 | VIALLGSIVIIVMNI | 53–67 |
| 135 | FTVFKDNEILYRAQL | 537–551 | 15 | LGSIVIIVMNIMIIQ | 57–71 |
| 136 | KDNEILYRAQLASED | 541–555 | 16 | VIIVMNIMIIQNYTR | 61–75 |
| 137 | ILYRAQLASEDTNAQ | 545–558 |  |  |  |
| 138 | AQLASEDTNAQKTIT | 549563 |  |  |  |
| 139 | SEDTNAQKTITNCFL | 553–567 |  |  |  |
| 140 | NAQKTITNCFLLKNK | 557–570 |  |  |  |
| 141 | TITNCFLLKNKIWCI | 561–575 |  |  |  |
| 142 | CFLLKNKIWCISLVE | 565–579 |  |  |  |
| 143 | KNKIWCISLVEIYDT | 569–582 |  |  |  |
| 144 | WCISLVEIYDTGDNV | 573–587 |  |  |  |
| 145 | LVEIYDTGDNVIRPK | 577–591 |  |  |  |
| 146 | YDTGDNVIRPKLFAV | 581–594 |  |  |  |
| 147 | DNVIRPKLFAVKIPE | 585–599 |  |  |  |
| 148 | RPKLFAVKIPEQCT | 589–603 |  |  |  |

^a^‘aa’ refers to amino acid.

**Supplementary Table 3. Accession Numbers of the Viral Sequences Used in the Sequence Analysis.**

| **Virus** | **Accession number** | **Country** | **Remarks** |
| --- | --- | --- | --- |
| Nipah virus (NiV) | NC_002728.1 | Malaysia | - |
|  | AY029767.1 | Malaysia | - |
|  | AY029768.1 | Malaysia | - |
|  | PQ463988.1 | Malaysia | - |
|  | KY425646.1 | Malaysia |  |
|  | KY425655.1 | Malaysia | - |
|  | MK673562.1 | Malaysia | - |
|  | PV165537 | Malaysia | Unpublished data |
|  | PV165539 | Malaysia | Unpublished data |
|  | PQ843374 | Malaysia | Unpublished data |
|  | PQ900425 | Malaysia | Unpublished data |
|  | PQ843362 | Malaysia | Unpublished data |
|  | PQ843363 | Malaysia | Unpublished data |
|  | PQ843364 | Malaysia | Unpublished data |
|  | PQ843365 | Malaysia | Unpublished data |
|  | PQ843366 | Malaysia | Unpublished data |
|  | PQ843367 | Malaysia | Unpublished data |
|  | PQ843368 | Malaysia | Unpublished data |
|  | PQ843369 | Malaysia | Unpublished data |
|  | PQ843370 | Malaysia | Unpublished data |
|  | PQ843371 | Malaysia | Unpublished data |
|  | PQ900424 | Malaysia | Unpublished data |
|  | PQ843372 | Malaysia | Unpublished data |
|  | PQ843373 | Malaysia | Unpublished data |
|  | PV165538 | Malaysia | Unpublished data |
|  | PV165536 | Malaysia | Unpublished data |
|  | AY988601.1 | Bangladesh | - |
|  | MK673564.1 | Bangladesh | - |
|  | MK673566.1 | Bangladesh | - |
|  | MK673567.1 | Bangladesh | - |
|  | MK673565.1 | Bangladesh | - |
|  | JN808857.1 | Bangladesh | - |
|  | JN808863.1 | Bangladesh | - |
|  | MK673568.1 | Bangladesh | - |
|  | JN808864.1 | Bangladesh | - |
|  | MK673570.1 | Bangladesh | - |
|  | MK673575.1 | Bangladesh | - |
|  | MK673576.1 | Bangladesh | - |
|  | MK673571.1 | Bangladesh | - |
|  | MK673572.1 | Bangladesh | - |
|  | MK673573.1 | Bangladesh | - |
|  | MK673574.1 | Bangladesh | - |
|  | MK673577.1 | Bangladesh | - |
|  | MK673578.1 | Bangladesh | - |
|  | MK673582.1 | Bangladesh | - |
|  | MK673579.1 | Bangladesh | - |
|  | MK673581.1 | Bangladesh | - |
|  | MK673583.1 | Bangladesh | - |
|  | MK673592.1 | Bangladesh | - |
|  | MK673589.1 | Bangladesh | - |
|  | MK673590.1 | Bangladesh | - |
|  | MK673591.1 | Bangladesh | - |
|  | MK673588.1 | Bangladesh | - |
|  | MK673585.1 | Bangladesh | - |
|  | MK673584.1 | Bangladesh | - |
|  | PP981665.1 | Bangladesh | - |
|  | PP981664.1 | Bangladesh | - |
|  | PP981667.1 | Bangladesh | - |
|  | PP981668.1 | Bangladesh | - |
|  | PP981670.1 | Bangladesh | - |
|  | PP981671.1 | Bangladesh | - |
|  | PP981669.1 | Bangladesh | - |
|  | PP981673.1 | Bangladesh | - |
|  | PP981675.1 | Bangladesh | - |
|  | PP981674.1 | Bangladesh | - |
|  | PP981676.1 | Bangladesh | - |
|  | PP981679.1 | Bangladesh | - |
|  | PP981682.1 | Bangladesh | - |
|  | PP981678.1 | Bangladesh | - |
|  | PP981681.1 | Bangladesh | - |
|  | PP981683.1 | Bangladesh | - |
|  | PQ368169.1 | Bangladesh | - |
|  | PQ368168.1 | Bangladesh | - |
|  | MH396625.1 | India | - |
|  | MH523640.1 | India | - |
|  | MH523641.1 | India | - |
|  | MH523642.1 | India | - |
|  | MK336155.1 | India | - |
|  | MK336156.1 | India | - |
|  | OR820506.1 | India | - |
|  | OR820508.1 | India | - |
|  | OR820507.1 | India | - |
| Hendra virus (HeV) | NC_001906.3 | Australia | - |

**Supplementary Table 4. Demographic, Clinical, and Exposure Information of NiV Survivors**

| **Survivor** | **Demographic information** | | | | | **Clinical symptoms at time of infection^b^** | **Hospitalized during outbreak** | **Long-term sequelae of NiV infection** | | | **Exposure to sick pigs in 1998-1999** | | **Cognitive assessment (MMSE^c^/MoCA^d^)** |
| --- | --- | --- | --- | --- | --- | --- | --- | --- | --- | --- | --- | --- | --- |
|  | **Age at infection** | **Current age^a^** | **Sex** | **Residence** | **Occupation in 1998-1999** |  |  | **Symptoms (if yes)** | **Onset of symptoms** | **Duration of symptoms** | **Close contact** | **Involved in handling/ processing of sick pigs** |  |
| S1 | 14 | 37 | M | Kampung Sungai Nipah | Pig farm helper | Yes | Yes | No |  |  | Yes | No | 30/30 |
| S2 | 28 | 51 | M | Kampung Bukit Pelanduk | Pig farmer | Yes | Yes | Seizures | After recovery from NiV infection | Until now | No | No | 26/20 |
| S3 | 23 | 46 | M | Kampung Bukit Pelanduk | Pig farmer | Yes | Yes | Poor eyesight | After recovery from NiV infection | Until now | Yes | Yes | 22/24 |
| S4 | 30 | 53 | M | Kampung Bukit Pelanduk | Pig farmer | Yes | Yes | No |  |  | Yes | No | 29/30 |

^a^Age at the time of questionnaire administration, January 2022.

^b^Clinical symptoms included fever, headache, dizziness, ataxia, vomiting, respiratory symptoms such as cough, and muscle pain.

^c^MMSE score interpretation: < 10 = severe cognitive impairment; 10–17 = moderate cognitive impairment; 18–26 = mild cognitive impairment; 27–30 = no cognitive impairment (normal).

^d^MoCA score interpretation: < 6 = severe cognitive impairment; 6–10 = moderate cognitive impairment; 11–25 = mild cognitive impairment; 26–30 = no cognitive impairment (normal).
